# The anti-idiotypic antibody 1F7 stimulates monocyte interleukin-10 production and induces endotoxin tolerance

**DOI:** 10.1186/1476-9255-10-14

**Published:** 2013-04-05

**Authors:** Tigran K Davtyan, David A Poghosyan, Anna G Sukiasyan, Michael D Grant

**Affiliations:** 1 Laboratory of Immunology and Virology, “Armenicum” Research Center CJSC, Yerevan, Armenia; 2 Institute of Molecular Biology NAS RA, Yerevan, Armenia; 3 Institute of Epidemiology MH RA, Yerevan, Armenia; 4 Division of BioMedical Sciences, Faculty of Medicine, Memorial University of Newfoundland, St. John’s, NL, Canada

**Keywords:** Hepatitis C virus, Monocyte, Interleukin-10, Idiotype, Endotoxin tolerance

## Abstract

**Background:**

Pathogens that establish chronic infection elicit immune responses with suppressive cytokines dominating over pro-inflammatory cytokines. Chronic hepatitis C virus (HCV) infection, human immunodeficiency virus (HIV) infection and simian immunodeficiency virus (SIV) infection are associated with high levels of antiviral antibodies expressing a common idiotype specifically recognized by the 1F7 monoclonal antibody (mAb). The 1F7 mAb is a murine IgMκ antibody raised against immunoglobulin pooled from the plasma of multiple HIV-infected individuals. In this study, we investigated direct effects of the 1F7 mAb itself on peripheral blood mononuclear cells (PBMC).

**Methods:**

Isolated monocytes or PBMC from healthy controls were incubated with the 1F7 mAb or IgMκ mAb control. Cytokine production was measured in cell culture supernatants by ELISA and cells producing interleukin-10 (IL-10) were identified by subset depletion and intracellular flow cytometry. Endotoxin tolerance was assessed by exposing monocytes to lipopolysaccharide (LPS) following 1F7 mAb or IgMκ mAb control pre-treatment and comparing tumor necrosis factor (TNF)-α levels in cell culture supernatants.

**Results:**

The 1F7 mAb stimulated monocytes and CD36^+^ lymphocytes to produce IL-10 in a time and dose-dependent manner. Treatment of monocytes with 1F7 mAb also reduced their subsequent responsiveness to LPS stimulation.

**Conclusions:**

Induction of antibodies expressing the 1F7 idiotype by chronic pathogens may facilitate IL-10 production and progression to chronic infection. Direct effects of IL-10 from human monocytes stimulated by 1F7-like antibodies, followed by monocyte transition to an alternatively activated phenotype illustrated by endotoxin tolerance, are two complementary features favouring a tolerogenic or non-responsive immunological environment.

## Background

Immunoglobulins contain unique primary sequences that are created by Ig gene rearrangement and further diversified by somatic hypermutation. The integrated composition of immunogenic epitopes within the variable (V) region of a given immunoglobulin (Ig) molecule is designated as its “idiotype” (Id). Anti-idiotypic antibodies reactive against determinants within the V regions of other antibodies may recognize rare “private” determinants, restricted to certain rearranged and modified sequences, or “public” determinants, common to germ-line sequences of components within the V region. Public or common idiotypes not associated with shared germ-line sequences arise through convergent selection processes. Antibodies with common idiotypes, but different antigenic specificity, usually emerge within a setting of chronic immune activation. In some cases, their selection may reflect adaptive exploitation of locally and systemically distributed idiotypic interactions by chronic pathogens [1,2]. The anti-idiotypic mAb 1F7 was raised in BALB/c mice injected with immunoglobulin pooled from multiple human immunodeficiency virus (HIV)-infected individuals [3]. The 1F7 idiotype is not restricted to any particular Ig heavy chain variable (VH) gene or gene family and occurs on human antibodies against (HIV) and hepatitis C virus (HCV) and on macaque antibodies against simian immunodeficiency virus (SIV) [4-7]. The 1F7 anti-idiotypic mAb reacts with human antibodies against HIV Env, Gag and Pol proteins, human antibodies against different HCV proteins and macaque antibodies against different SIV proteins [4-8]. This broad distribution of the 1F7-defined idiotope on parallel sets of antibodies against different chronic pathogens positions it at a common idiotypic convergence point connected to chronic infection or immune activation.

An association between the level of antibodies expressing the 1F7 Id and outcome of infection was recently shown in hepatitis C infection. The level of antibodies reacting with the anti-idiotypic mAb 1F7 is significantly higher in persons with chronic hepatitis C infection than in either healthy donors or in persons who spontaneously cleared HCV [9]. The 1F7 Id is also more common on the Ig B cell receptors of CD5^+^ B1 B cells than on the Ig B cell receptors of CD5^-^ B cells. Since B1 B cells are a source of interleukin-10 (IL-10), we speculated that interactions between HCV proteins and B1 B cells that drive production of 1F7 Id-expressing anti-HCV antibodies might also stimulate IL-10 production by B1 B cells and monocytes during HCV infection [10,11].

In acute HCV infection, production of pro-inflammatory TH1-type cytokines by T cells in response to viral antigens correlates with self-limited infection, while production of TH2-type cytokines heralds chronic infection [12,13]. Similarly, chronic HCV infection is marked by elevated TH2-type and reduced TH1-type cytokine responses [14,15]. Peripheral TH1-type cytokines rise in parallel with virological responses to interferon-alpha (IFN-α) therapy [16,17], while TH2-type cytokines fall together with viral load [18,19]. The amount of IL-10 induced during acute infection is a critical determinant of progression to chronic infection versus viral clearance in certain animal model systems and induction of IL-10 by HCV proteins has been demonstrated in several in vitro studies [20,21]. Subjects with untreated chronic HCV infection have elevated serum levels of IL-10 and disease association studies of IL-10 promoter polymorphisms indicate that IL-10 levels are important in both susceptibility to infection and resistance to inflammatory disease [22].

Here we studied effects of the anti-idiotypic mAb 1F7 on IL-10 production by normal human peripheral blood monocytes and CD36^+^ lymphocytes. We previously showed that CD36^+^ lymphocytes (CD14-negative non-T, non-B non-NK cells with lymphocyte forward and side scatter characteristics by flow cytometry) constitutively produce IL-10 and potentially influence homeostatic innate immune suppression [23]. We also studied the influence of 1F7 mAb on IL-10 and tumor necrosis factor-α (TNF-α) production by isolated monocytes activated by toll-like receptor 4 (TLR4) and nuclear oligomerization domain (NOD)-like (agonists), and the influence of 1F7 mAb on monocyte endotoxin tolerance.

## Methods

### Influence of 1F7 mAb on IL-10 production by peripheral blood mononuclear cells (PBMC)

The 1F7 mAb was produced as murine hybridoma culture supernatant with IgM concentration measured by ELISA [7]. Peripheral blood samples were obtained from 10 healthy donors (4 male and 6 female). Approval for this study was obtained from the Ethics Committee of Yerevan State Medical University and informed consent was obtained from all donors. Peripheral blood mononuclear cells (PBMC) were collected by histopaque-1077 (Sigma-Aldrich) gradient centrifugation of whole blood and resuspended in RPMI-1640 medium containing 10% fetal calf serum (FCS), 5 mM HEPES, 2 mM L-glutamine, 1 mM sodium pyruvate, 100 U penicillin, and 100 μg streptomycin/ml (all from Invitrogen). 2.5 × 10^6^ PBMC were treated with 0.48-1.92 μg/ml mAb 1F7 by two-fold dilution of 1F7 hybridoma supernatant or with an IgMκ mAb control (eBioscience) at equivalent IgM concentrations. The endotoxin content in 1F7 hybridoma supernatant or IgMκ control was determined by Limulus Amebocyte Lysate assay (Pyrogent, Lonza), with detection limit 0.03 EU/ml, as per the manufacturer’s recommendations. Cells were incubated for 24, 48 and 72 h with 1F7 mAb or IgMκ control at 37°C with 5% CO_2_. After incubation, cells were pelleted by centrifugation and the supernatants collected and stored at −80°C until cytokines were measured. The amount of IL-10 in supernatants was determined as per the manufacturer’s instructions by ELISA using the human IL-10 Ready-SET-Go test kit (eBioscience) with a detection limit of 2 pg/ml.

### Influence of 1F7 mAb on IL-10 production by purified monocytes

Peripheral blood monocytes were isolated by adherence of isolated PBMCs to 25 cm^2^ plastic flasks for 45 min at 37°C in 6% CO_2_[24]. Adherent monocytes were washed three times with endotoxin-free PBS and cultured at 5 × 10^5^ cells/ml in complete medium. Lipopolysaccharide (LPS) from *E. coli 026:B6* at 100 ng/ml or 2.5 μg/ml peptidoglycan (PGN), a NOD1 and NOD2 ligand, from *E. coli 0111:B4* (both from Invivogen) were included in the culture media from day 0 to day 3. Cell free supernatants collected at days 1 and 3 were stored at −80°C until IL-10 was measured. Monocytes were depleted from PBMC using EasySep® human CD14 magnetic nanoparticles (Stemcell) according to the manufacturer’s instructions. The purity of positively selected monocytes, as assessed by cell surface staining with fluorescein isothiocyanate (FITC)-conjugated anti-CD14 (eBioscience, clone 61D3, IgG1κ) and flow cytometry, ranged from 97.5-98.3%.

### Intracellular and cell surface staining of monocytes and lymphocytes

After PBMC incubation with the 1F7 mAb, 5 × 10^5^ cells were surface stained with either 0.3 μg (FITC)-conjugated anti-CD36 (Serotec, clone SMO, IgM), anti-CD14-FITC or isotype-matched controls for 30 min at room temperature and fixed in 1% paraformaldehyde (Becton Dickinson). Stained cells were incubated with 1% FACS permeabilizing solution (Becton Dickinson), followed by phycoerythrin (PE)-conjugated anti-human IL-10 or IL-4 (eBioscience, clones JES3-9D7, IgG1κ and 8D4-8, IgG1κ respectively) or isotype-matched controls and 5000 gated events were analysed on a FACSCalibur™ instrument with CellQuest software (Becton Dickinson). Instrument set up and colour compensation was done using BD Calibrite™ beads and FACSComp software. Apoptosis was monitored by measuring the proportion of Annexin V-positive, PI-negative cells using PI/Annexin V kits (BD PharMingen) [25].

### Assessment of endotoxin tolerance

Endotoxin tolerance was monitored by measuring production of TNF-α and IL-10 by monocytes in response to LPS after preincubation with LPS or mAb 1F7 [26]. Isolated monocytes at 5 × 10^5^ cells/ml were cultured for 18 h with 100 ng/ml LPS, 1.92 μg/ml mAb 1F7 or in plain medium. After 18 h of culture, the monocytes were washed three times with endotoxin-free PBS, and cultured for an additional 4 h with 1 μg/ml LPS. Incubation of monocytes with 100 ng/ml LPS, followed by rechallenge with 1 μg/ml LPS is a standard method for assessing endotoxin tolerance [25,26]. At the end of the culture period, cell-free supernatants were collected and endotoxin tolerance assessed by measuring human TNF-α and IL-10 with Ready-SET-Go test kits (eBioscience) according the manufacturer’s instructions.

### Statistical analysis

Data from independent experiments were used to calculate mean values ± SE and differences were tested for statistical significance (p ≤ 0.05) by Student’s paired *t* test. ANOVA for repeated measures was used for analysis of mAb 1F7 action on endotoxin tolerance induction and cytokine production.

## Results

### The 1F7 mAb induces IL-10 production by PBMC in vitro in a dose- and time-dependent manner

**Figure 1:**
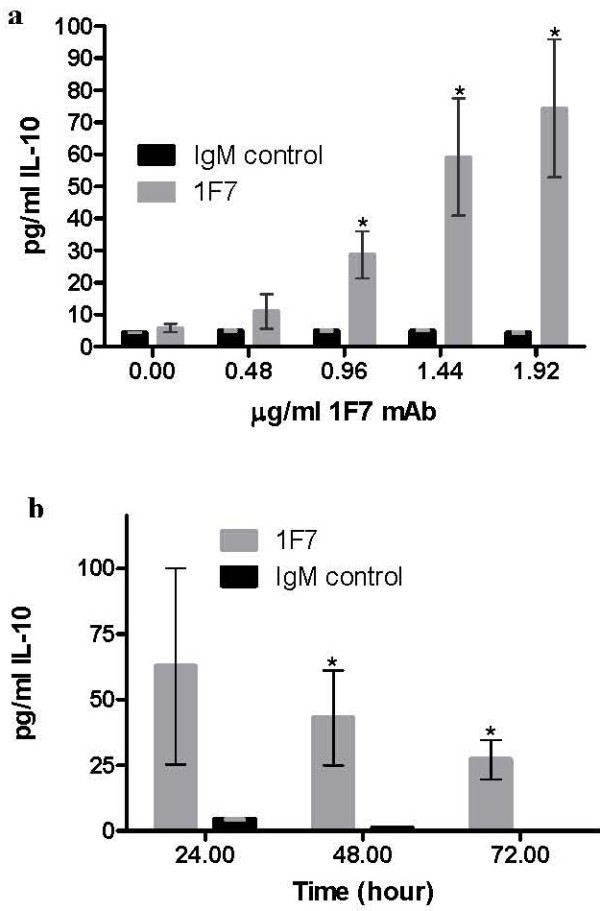
**Time and dose-dependent effects of 1F7 mAb on IL-10 production by PBMC in vitro.** PBMC from 10 individuals were incubated with 0.48-1.92 μg/ml 1F7 mAb or IgMκ control mAb for 18 h (**a**) or with 1.92 μg/ml mAb 1F7 or control for 24, 48 and 72 h (**b**). The IL-10 concentration in cell free supernatants was then measured by ELISA. Data shown represent mean values ± SE. Mean IL-10 levels significantly different between 1F7 mAb-treated and IgMκ mAb control-treated cell supernatants are noted (*p < 0.05).

**Figure 2:**
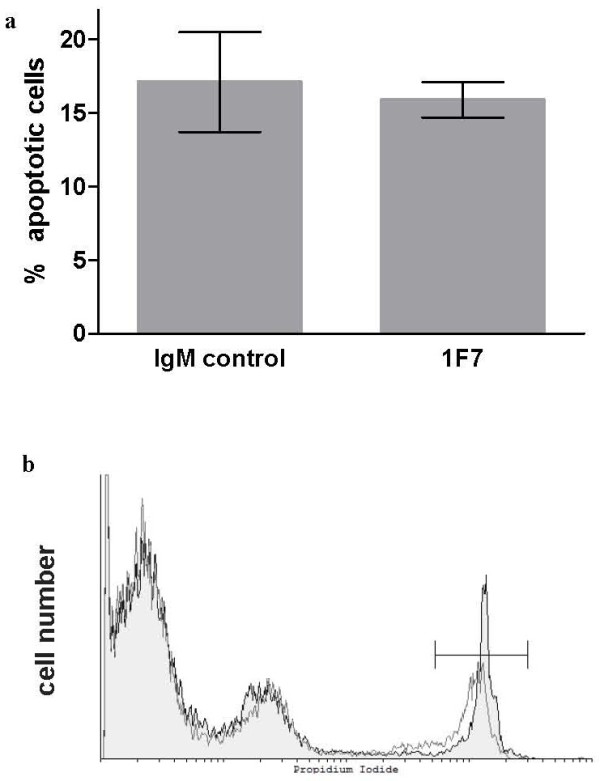
**Effect of 1F7 mAb on PBMC apoptosis and necrosis.** PBMC were incubated with 1.92 μg/ml 1F7 mAb or IgMκ mAb control for 72 h and analyzed by flow cytometry for Annexin V^+^, propidium iodide (PI)^-^ apoptotic cells (**a**) or PI^+^ necrotic cells (**b**). Data shown in (**a**) represent mean values ± SE, n = 10. The shaded area of a representative flow cytometry plot (**b**) corresponds to IgMκ control mAb-treated cells (13.9% PI^+^) and the non-shaded area corresponds to 1F7 mAb-treated cells (12.0% PI^+^).

We first tested whether 1F7 mAb stimulated IL-10 production by normal human PBMC in vitro. Freshly isolated PBMC from 10 healthy donors were incubated individually for 18 h with 0.48, 0.96, 1.44 and 1.92 μg/ml 1F7 mAb, after which IL-10 was measured in culture supernatants. The starting concentration of IgM mAb in the hybridoma supernatant was 1.92 μg/ml, therefore, dose response was titrated from this level. We found that 1F7 mAb at 0.96, 1.44 and 1.92 μg/ml induced significant (p = 0.01) IL-10 production in PBMC with responses increasing in a dose-dependent manner (Figure 1a). The timing of 1F7 mAb stimulation of IL-10 production was studied at 1.92 μg/ml 1F7 mAb because this concentration induced the most IL-10 secretion. 1F7 mAb-treated cells were incubated for 24, 48 and 72 h, after which the IL-10 concentration in culture supernatants was measured. These time course studies (Figure 1b) showed maximal IL-10 concentration at 24 h (62.7 ± 37.4 pg/ml), with a gradual decrease at 48 h (43.1 ± 17.9 pg/ml) and 72 h (27.1 ± 7.5 pg/ml) (p = 0.03). To test whether the decreasing IL-10 production over time reflected effects of 1F7 mAb on cell survival, we assessed apoptotic and necrotic cell death by flow cytomtery. The results indicate that 1F7 mAb does not cause apoptotic (Figure 2a) or necrotic (Figure 2b) cell death. Thus, secretion of IL-10 by PBMC dramatically increases shortly after treatment with 1F7 mAb, but then decreases as incubation extends past 24 h.

### 1F7 mAb induces IL-10 production by CD14^+^ monocytes and CD36^+^ lymphocytes

**Figure 3:**
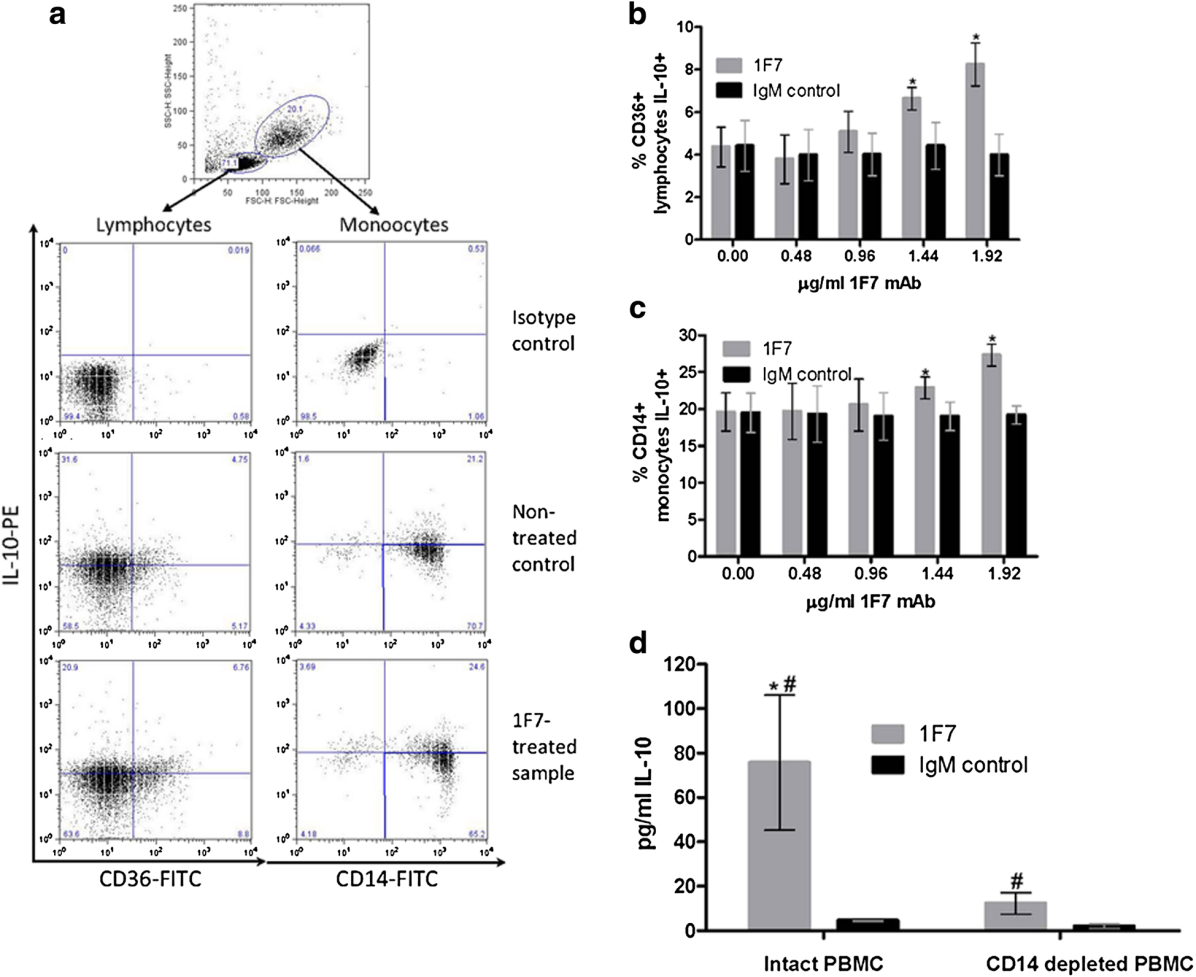
**Identification of PBMC subsets producing IL-10 in response to IF7 mAb.** PBMC from 10 healthy donors were surface stained with anti-CD36 or anti-CD14, fixed, permeabilized and incubated with anti-human IL-10 or matched isotype control. CD36^+^ lymphocytes and CD14^+^ monocytes were analyzed for intracellular IL-10 as shown (**a**). Bar graphs show mean values ± SE (**b**-**c**). Significant differences between stimulation with mAb 1F7 and the IgMκ control mAb control are indicated (*p < 0.05). For 5 individuals, CD14^+^ monocytes were depleted and the remaining cells incubated with 1.92 μg/ml 1F7 mAb or IgMκ control mAb for 24 h, after which IL-10 in culture supernatants was measured (**d**). The mean IL-10 concentration in supernatant was significantly different between 1F7 mAb-treated, monocyte-depleted PBMC and intact PBMC (*p < 0.05) and between 1F7 mAb and IgMκ control mAb-treated monocyte-depleted PBMC (#p < 0.02).

Next, we studied which PBMC subsets produce IL-10 in response to 1F7 mAb. Freshly isolated PBMC from the same 10 healthy donors were incubated with 0.48, 0.96, 1.44 and 1.92 μg/ml 1F7 mAb for 18 h and then surface stained for CD14 and CD36, permeabilized, stained for intracellular IL-10 and analyzed by flow cytometry (Figure 3a). We found that in a dose-dependent manner, 1F7 mAb significantly increased the percentage of CD36^+^ lymphocytes (p = 0.02) and CD14^+^ monocytes (p = 0.03) producing IL-10 (Figure 3b, c). To determine the major source of IL-10 production in response to 1F7 mAb, we next compared production of IL-10 by CD14^+^ monocyte-depleted and intact PBMC from 5 of the 10 healthy donors. CD14^+^ cells were depleted using magnetic beads and the remaining cells incubated with 1.92 μg/ml 1F7 mAb or IgM control for 24 h, after which the IL-10 concentration in culture supernatants was measured. As shown in Figure 3d, intact PBMC from the 5 selected healthy donors incubated with 1F7 mAb produced similar amounts of IL-10 (75.6 ± 30.4 pg/ml) to the amounts previously produced by the 1F7 mAb stimulated PBMC of all 10 healthy donors (62.7 ± 37.4 pg/ml, Figure 1). Depletion of CD14^+^ monocytes dramatically decreased IL-10 production (Figure 3d). However, the 1F7 mAb still induced significantly more IL-10 production by CD14^+^ monocyte-depleted PBMC than was induced by the IgMκ mAb control (12.2 ± 4.8 pg/ml versus 1.99 ± 0.98 pg/ml, p = 0.02, Figure 3d).

### Time-dependent 1F7 mAb stimulation of cytokine production by PBMC subsets

**Figure 4:**
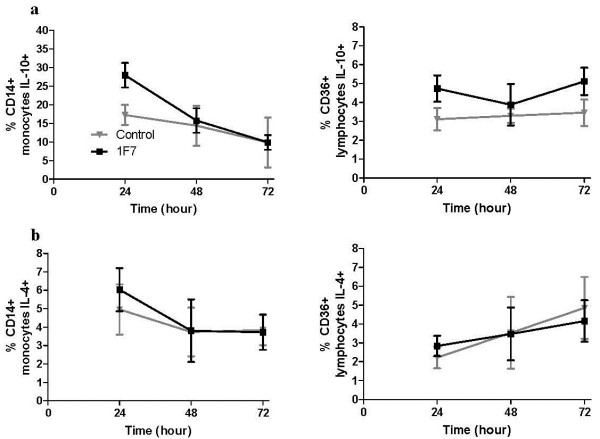
**Time-dependent effects of 1F7 mAb on IL-4 and IL-10 production by CD14**^**+ **^**monocytes and CD36**^**+ **^**lymphocytes.** PBMC from 10 healthy donors were incubated with 1.92 μg/ml 1F7 mAb or IgMκ mAb control for 24, 48 and 72 h and surface stained with anti-CD36 or anti-CD14. Cells were then fixed, permeabilized, incubated with anti-human IL-10, anti-IL-4 or isotype-matched controls and analyzed by flow cytometry for intracellular IL-10 (**a**) and IL-4 (**b**). Gating for intracellular cytokine analysis was done as shown in Figure 3. Data shown represent mean values ± SE.

Next, we studied the time-dependent action of 1F7 mAb on IL-4 and IL-10 production by normal human monocytes and lymphocytes. Freshly isolated PBMC were incubated with 1.92 μg/ml 1F7 mAb or IgMκ control mAb for 24, 48 and 72 h and the percentage of monocytes and CD36^+^ lymphocytes producing IL-4 or IL-10 analyzed by flow cytometry. Similar to the time course studies carried out with PBMC (Figure 1b), 1F7 mAb significantly increased the percentage of IL-10 producing monocytes at 24 h (p = 0 .001), with a gradual decrease at 48 h and 72 h (Figure 4a). In contrast, 1F7 mAb significantly increased the percentage of IL-10 producing CD36^+^ lymphocytes at both 24 h (p = 0.005) and 72 h (p = 0.002). The temporal pattern of 1F7 mAb action on IL-4 production by CD14^+^ monocytes was similar to its action on IL-10 production with a peak at 24 hours and gradual decrease at 48 and 72 hours (Figure 4b). In contrast to 1F7 mAb’s action on IL-10 production by both monocytes and lymphocytes, we saw no statistically significant differences in IL-4 production between 1F7 mAb and IgMκ control mAb treated cells over the 24–72 h incubation period (Figure 4a, b). Thus, we conclude that 1F7 mAb selectively induces short-term production of IL-10 by monocytes, which begins to decline after 24 hours.

### 1F7 mAb enhances TLR and NOD agonist-induced IL-10 production by monocytes

**Figure 5:**
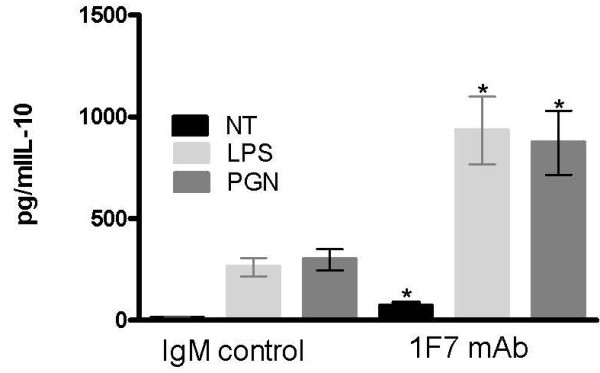
**Impact of 1F7 mAb on TLR and NOD agonist-induced IL-10 production by monocytes.** Peripheral blood monocytes obtained from 10 healthy donors were unstimulated (NT) or stimulated with either 100 ng/ml LPS or 2.5 μg/ml PGN for 3 days in the presence of 1.92 μg/ml 1F7 mAb or IgMκ control mAb. Supernatant IL-10 concentration was then measured. All data shown represent mean values ± SE and significant differences between 1F7 mAb-treated and IgMκ control mAb-treated monocytes are noted (*p < 0.05).

Recognition of pathogen-associated molecular patterns by TLRs and NOD proteins is an important initial step in mounting an immune response against bacteria and some viruses. Therefore, we investigated how 1F7 mAb influenced production of IL-10 by isolated monocytes treated with TLR and NOD agonists. TLR agonist LPS and NOD agonist PGN were added to isolated monocytes together with 1F7 mAb or IgMκ control mAb at 1.92 μg/ml for 72 h and IL-10 production was measured in culture supernatants (Figure 5). We found that 1F7 mAb significantly increased IL-10 production by untreated (NT) monocytes and augmented IL-10 production by LPS- and PGN-stimulated monocytes (p < 0.05). These results were somewhat unexpected as it was shown above that 1F7 mAb dramatically increases production of IL-10 by monocytes at 24 h, but then the concentration of IL-10 decreases at 48 and 72 h.

### 1F7 mAb induces monocyte endotoxin tolerance

**Figure 6:**
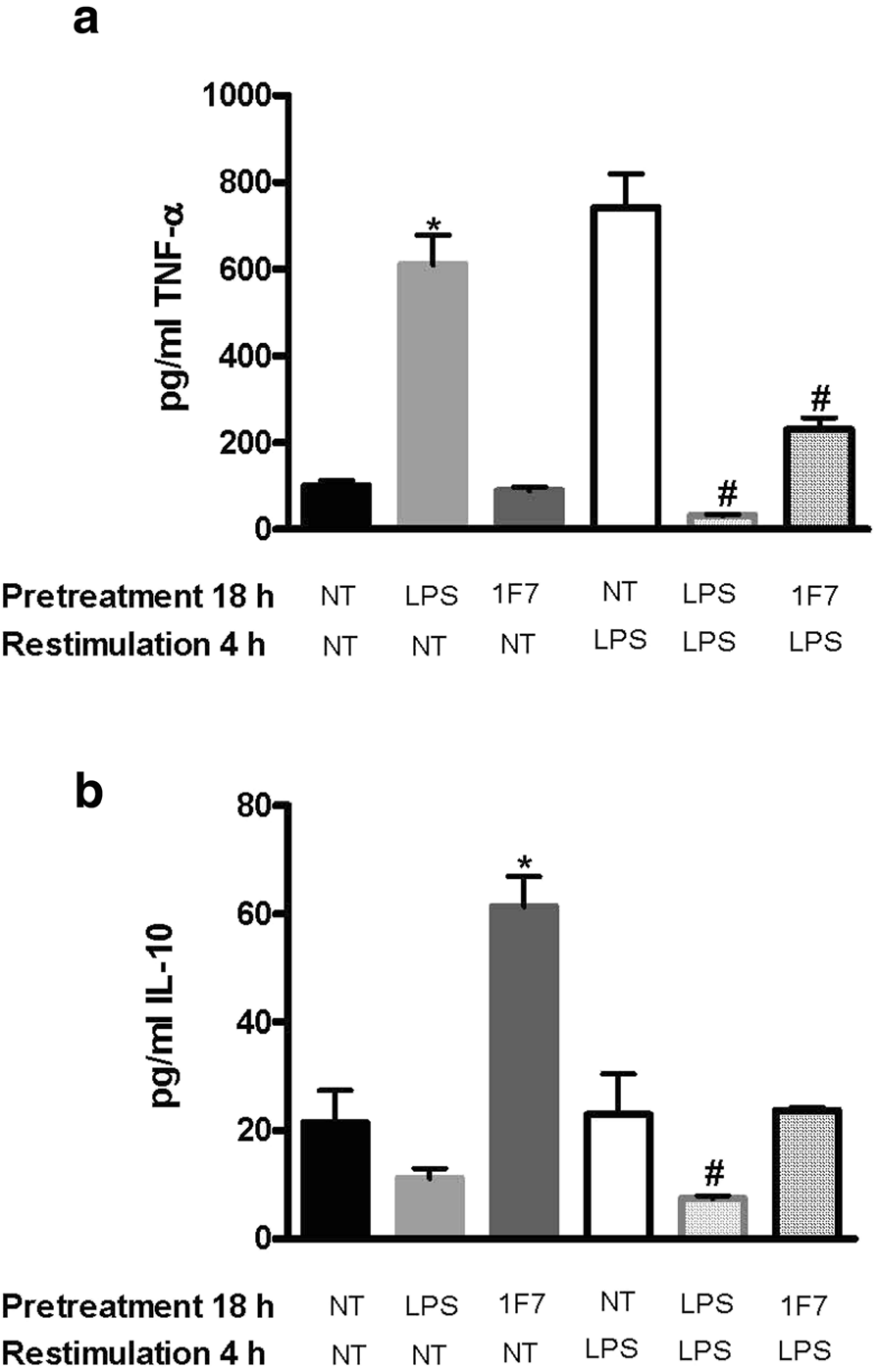
**Influence of 1F7 mAb treatment on monocyte endotoxin tolerance.** Monocytes from 10 healthy donors were pre-treated with 100 ng/ml LPS or 1.92 μg/ml 1F7 mAb for 18 h, washed with LPS-free PBS and incubated for an additional 4 h with 1 μg/ml LPS or 1.92 μg/ml 1F7 mAb. Levels of TNF-α (**a**) and IL-10 (**b**) in supernatants were then measured. All data shown represent mean values ± SE. Significant differences by repeated ANOVA measures are noted between NT, LPS or 1F7 treatment (*p < 0.05) and between LPS re-stimulated pairs (#p < 0.05).

To determine whether 1F7 mAb-induced production of IL-10 is associated with anti-inflammatory (alternative) activation of monocytes, we next studied LPS tolerance induction *in vitro*. Monocytes were pretreated with LPS at 100 ng/ml or 1F7 mAb at 1.92 μg/ml for 18 h, after which the cells were washed with LPS-free PBS and restimulated with LPS or 1F7 for another 4 h. Production of TNF-α and IL-10 was subsequently measured in cell culture supernatants. We found that at 18 h incubation, LPS, but not 1F7 mAb, stimulated monocyte TNF-α production (Figure 6a). This is consistent with 1F7 mAb causing early anti-inflammatory (alternative) activation of unstimulated monocytes. As expected, the monocytes developed homologous tolerance to LPS challenge shown by declining production of TNF-α after overnight LPS treatment (Figure 6a, NT+LPS versus LPS+LPS treatment groups). Similar to the LPS-treated monocytes, monocytes treated overnight with 1F7 mAb also developed tolerance to subsequent LPS challenge as shown by a statistically significant decline in production of TNF-α (Figure 6a, NT+LPS versus 1F7 mAb+LPS treatment groups, p < 0.002).

Next, we studied how monocytes responded in terms of IL-10 production to overnight LPS treatment (Figure 6b). We found that 18 h incubation with 1F7 mAb, but not LPS induced monocyte IL-10 production (Figure 6b), consistent with 1F7 mAb inducing early anti-inflammatory (alternative) activation of monocytes. LPS-pretreated monocytes produced a low, but detectable level of IL-10 following repeated LPS stimulation (Figure 6b, p < 0.04). Overnight culture of monocytes with either no treatment or with 1F7 mAb treatment prior to LPS restimulation resulted in a significantly higher level of IL-10 compared to repeated LPS treatments (Figure 6b, p < 0.04).

## Discussion

Cytokine production profile is closely associated with the outcome of viral infection. A predominance of pro-inflammatory TH1-type cytokines such as IL-12 and IFN-γ portends viral clearance or control, whereas dominance of anti-inflammatory cytokines like IL-4 or IL-10 is more likely to herald chronic infection [12-15]. This suggests that pathogens establishing chronic infection have evolved mechanisms to skew host responses towards cytokine profiles that favour their persistence. In a number of infections, the level and timing of IL-10 production is a pivotal factor in determining pathogen clearance versus pathogen persistence [27-30]. We previously demonstrated an association between development of chronic HCV infection, the level of anti-HCV antibodies expressing a common idiotype recognized by the 1F7 mAb and expansion of B1 B cells expressing the same idiotype [9]. Therefore, we speculated that HCV may exploit a link between B1 B cell activation, induction of 1F7 Id-expressing antibodies and IL-10 production to evade the immune system and establish chronic infection.

In this study, we found that the 1F7 mAb itself specifically triggered IL-10 production by freshly-isolated PBMC in a time and dose-dependent manner. Both CD36^+^ lymphocytes and CD14^+^ monocytes produced IL-10 in response to 1F7 mAb. Although the percentage of CD36^+^ lymphocytes producing IL-10 doubled following 1F7 mAb treatment, in absolute terms this was a small number of responding cells compared to the number of monocytes producing IL-10 in response to 1F7 mAb treatment. Monocytes generally represent ~20% of total PBMC, while CD36^+^ lymphocytes represent < 1% of the lymphocyte population. Depletion of CD14^+^ monocytes reduced 1F7 mAb-stimulated IL-10 production by > 80%, therefore, we concluded that CD36^+^ lymphocytes are a minor source of IL-10 production following 1F7 mAb stimulation. The initial induction of monocyte IL-10 production by 1F7 mAb was followed by imposition of classical endotoxin tolerance in that the pro-inflammatory response to TLR ligands such as LPS was substantially blunted. If these in vitro responses to the 1F7 mAb itself reflect responses that occur in vivo following activation of B1 B cells bearing Ig with the 1F7 idiotype, this could represent a two-pronged approach for pathogens to suppress immune responses that favour clearance. Since the idiotype recognized by mAb 1F7 is more common on CD5^+^ B1 B cells and these cells produce IL-10 [9-11], we initially felt that 1F7 interacting in vitro with the Ig B cell receptor of B1 B cells might mimic in vivo interactions with HCV proteins that directly trigger IL-10 production. However, the major source of IL-10 following exposure to 1F7 mAb is monocytes, not B cells, indicating that the IL-10 is not produced as a direct effect of 1F7 mAb binding to the Ig B cell receptor of B1 B cells. While the mechanism by which the 1F7 mAb itself stimulates monocyte production of IL-10 in vitro is non-antigen specific, it may represent a mechanism by which HCV and other chronic viral and bacterial pathogens selectively exploit idiotypic connections to suppress immune responses. It was recently shown that by differentially affecting TLR4 and TLR8 pathways, IL-10 may selectively modulate monocyte functions in persons with chronic HCV infection [31]. This corroborates our data suggesting that IL-10-mediated inhibition of the TLR4 signaling pathway in monocytes induces endotoxin tolerance and favours alternative activation of monocytes [31].

Convergent selection of anti-HCV antibodies bearing the 1F7 idiotype occurs during HCV infection and involves activation of B1 B cells [9]. Due to the high degree of V region connectivity between B1 B cells, they may be activated either by direct interaction with HCV proteins or through interaction with other antibodies simulated by HCV [32,33]. Since the 1F7 mAb is a multimeric IgM mAb, its overall high avidity may produce exaggerated effects in vitro compared to IgG antibodies. However, the high V region connectivity and high frequency of expression of the 1F7 idiotype on B1 B cells suggests that immune complexes containing 1F7 Id-expressing and 1F7-like antibodies will form when B1 B cells are activated. If so, these complexes will have similar overall avidity to the 1F7 IgM mAb. These complexes or the 1F7-like antibodies themselves [8], should act on circulating monocytes during acute HCV infection in the same way as the 1F7 mAb acts in vitro, by inducing IL-10 production and endotoxin tolerance. Suppression of proinflammatory cytokines such as IFN-γ, and IL-12 by monocyte-derived IL-10 induced in this manner would also favour chronic HCV infection. Thus, early activation of B1 B cells producing Ig expressing the 1F7 idiotype and B1 B cells with complementary 1F7 Id-like Ig receptors could underlie the association we observed between high levels of antibodies expressing the 1F7 idiotype and chronic HCV infection [9]. Stimulation of 1F7 Id-expressing antibodies by chronic pathogens susceptible to phagocytosis and intracellular killing could favour their persistence through both the direct IL-10 production and subsequent endotoxin tolerance imposed on monocytes.

The idea that pathogens can exploit idiotypic interactions inherent to the humoral immune system was initially proposed by Irun Cohen [34-36]. Common idiotypes arise in the setting of chronic infection or chronic immune activation and are often carried on antibodies termed natural antibodies [37]. Natural antibodies are produced by B1 B cells and demonstrate cross-reactivity, autoreactivity and high V region connectivity [38-40]. Activating immune responses along this axis of complementary autoreactive and cross-reactive idiotypic dominance would in itself benefit pathogens in that the response would be more likely to remain diffuse. If activating immune responses along this axis also leads to IL-10 production and monocyte endotoxin tolerance, prospects for establishing chronic infection would be further enhanced. In this regard, all of the pathogens so far shown to stimulate antibodies expressing the 1F7 idiotype either predominantly (HCV) or uniformly (HIV and SIV) establish chronic infection. This suggests that chronic pathogens have evolved to exploit inherent immune system structural characteristics such as high connectivity and functional coupling to IL-10 induction, which are better suited to preserve self-tolerance than to provide protection against pathogens.

## Conclusions

Our previous study showed an association between 1F7 Id expression levels and chronic HCV infection [8]. In the present study, we investigated the potential role of antibodies bearing the 1F7 Id in modulating monocyte cytokine responses. We observed that the mAb 1F7 itself induced IL-10 production by unstimulated and TLR- or NLR-agonist-stimulated monocytes and subsequently imposed endotoxin tolerance on those monocytes. However, pretreatment of monocytes with LPS followed by LPS was significantly different from pretreatment with 1F7 mAb followed by LPS in terms of both TNF-α production and IL-10 production. This indicates that monocyte endotoxin tolerance imposed by 1F7 mAb treatment is incomplete compared to LPS-induced monocyte endotoxin tolerance. The 1F7 mAb probably provides a weaker primary stimulus than LPS and acts through a different signaling pathway. A distinct or divergent signaling pathway from LPS is also suggested by the lack of significant TNF-α production in response to 1F7 mAb. Although the receptor on monocytes that mediates 1F7 mAb signaling is unknown, the effects are selective and specific. Further elucidation of the immunological and biochemical basis for the association between 1F7 Id expression levels and chronic HCV infection and for the selective action of 1F7 mAb on monocytes may aid in the design of novel therapeutic and prophylactic strategies against HCV and other chronic pathogens.

## Competing interests

Michael Grant is a member of the Scientific Advisory Board of Network Immunology Inc., a private company developing vaccines based on the 1F7 mAb.

## Authors’ contributions

MDG and TKD carried out the study design, project oversight, data analysis and manuscript preparation. DAP and AGS carried out sample collection, flow cytometry, data analysis and manuscript preparation. DAP also contributed to study design, and oversaw the lipopolysaccharide tolerance experiments. All authors read and approved the final manuscript.
